# Comparison of the Ways in Which Nitidine Chloride and Bufalin Induce Programmed Cell Death in Hematological Tumor Cells

**DOI:** 10.1007/s12010-023-04468-z

**Published:** 2023-04-22

**Authors:** Zejie Su, Man Luo, Zhi Lian Chen, Hai Lan

**Affiliations:** 1 Department of Pharmacy, Shunde Hospital of Guangzhou University of Chinese traditional Medicine, Shunde, People’s Republic of China; 2 Department of Hemalology, the First Affiliated Hospital of Guangzhou University of Chinese Medicine, Guangzhou, People’s Republic of China

**Keywords:** Bufalin, Jurkat cells, Nitidine chloride, Programmed cell death(PCD), RPMI-8226 cells, THP-1 cells

## Abstract

The objective of this work to study the programmed cell death (PCD) in hematological tumor cells induced by nitidine chloride (NC) and bufalin (BF). Hematological tumor cells were exposed to various doses of NC and BF to measure the level of growth inhibition. While inverted microscope is used to observe cell morphology, western blot technique is used to detect apoptosis-related protein expression levels. The effects of NC and BF on hematological tumor cells were different. Although abnormal cell morphology could be seen under the inverted microscope, the western blot results showed that the two medicines induced PCD through different pathways. Drug resistance varied in intensity across distinct cells. THP-1, Jurkat, and RPMI-8226 each had half maximum inhibitory concentrations (IC50) of 36.23 nM, 26.71 nM, and 40.46 nM in BF, and 9.24 µM, 4.33 µM, and 28.18 µM in NC, respectively. Different hematopoietic malignancy cells exhibit varying degrees of drug resistance, and the mechanisms by which apoptosis of hematologic tumor cells is triggered by NC and BF are also distinct.

## Introduction

Blood-related cancers frequently start in the bone marrow, which makes blood. White blood cells, red blood cells, or platelets can be produced by bone marrow stem cells [1–3]. When the uncontrolled development of irregular blood cells outpaces the growth of healthy blood cells and interferes with their ability to function normally, hematologic malignancies result. Malignant tumors that develop in the blood system which consist of bone marrow, hematopoietic tissue, and lymphoid tissue are called hematologic malignancies [4, 5]. Hematologic cancers are defined by the World Health Organization (WHO) as malignancies of the lymphatic, bone marrow, histocyte, and dendritic cell lineages. Because these tumor cells tend to congregate in the liver, spleen, lymph nodes, and other hematological organs, chemotherapy is still the go-to therapeutic option. Therefore, it is important to find a chemotherapy regimen that is indeed effective for hematological tumors.

Although an immunotherapy is developed this year, it is difficult to promote it due to the high cost of treatment. Nitidine chloride, as a benzophen anthridine alkaloid, is a natural alkaloid obtained from the dried roots of *nitidum* that is a species of rare rutaceae plant native to Guangxi, China. According to previous reports, NC has a wide range of antitumor activities and significant inhibitory effects on digestive, respiratory, reproductive, breast, and other tumors [6, 7]. Bufalin is a cardiotonic steroid isolated from the venom called Chansu of *bufo chinensis* [8–10]. In Chinese medicine, it is a Galen preparation of the dried white venom of the Chinese toad (*Asian toad*). The molecular formula of BF is C_24_H_34_O_4_, and the relative molecular weight is 386.5 g/mol. As an active compound extracted from traditional Chinese medicine, BF has a variety of biological effects, including pain relief, stimulation of myocardial contraction, stimulation of blood pressure, anti-inflammatory, and anti-tumor activity [11]. Both NC and BF can effectively inhibit tumor cell proliferation and promote PCD [12, 13]. Although the precise efficacy and regulatory mechanism are yet unknown, it is often employed in clinical applications because of its efficacy.

This study compares the efficiency of the two drugs in the treatment of several types of hematological malignancies, which has yet to be completely explored, as well as a preliminary examination of the molecular processes of apoptosis. Multiple myeloma, malignant lymphoma, and various types of leukemia are the most prevalent hematological cancers. However, it is unclear if both medications are beneficial in all of these tumors or whether the two may be taken in combination to improve efficacy. To make the greatest use of the two medications and avoid drug resistance, Jurkat, THP-1, and RPMI-8226 cells were chosen to try to understand the difference in treatment efficacy by identifying apoptotic proteins.

## Materials and Methods

### Methods

#### Cell Culture

All cell lines were obtained from the Cell Bank of the Representative Culture Preservation Committee of the Chinese Academy of Sciences. All the cells were cultured in RPMI-1640 (Gbico™, 11,875,119) supplemented with 10% FBS (Gbico™, 10,099,141 C) at 37 °C in 5% CO_2_ and subcultured every 2–3 days until the cells entered the logarithmic growth phase.

#### Detection of Inhibition Rate of Cell Proliferation

NC (Topscience, T5S0761) and BF (Topscience, T1719) were mixed with dimethyl sulfoxide at the ratio of 100:1, fully dissolved, diluted to 1 mg/mL, filtered and sterilized, and stored in a refrigerator at 4℃. Centrifuge cells that are in the logarithmic growth phase at a low speed of 800 r/min for 3 min, remove the culture medium, then add 100 µL of RPMI-1640 culture medium, and gently pipette to resuscitate the cells. Cell counter was used to count the cells, then seed 96-well plates with 5000 cells each well, repeating three groups. At the end of the incubation period, 10 µL CCK-8 reagent (Beyotime, C0039) was added to each well and incubated at 37 °C for 1 h according to the manufacturer’s instructions. After staining, the absorbance was measured at microplate reader (Thermofish, MULTISKAN FC 51,119,080). The absorbance wavelength was 450 nm, while the 630 nm wavelength was chosen for dual-wavelength detection. Inhibition rate of cell proliferation (%) = [(control group - experimental group) / (control group - blank control group)] × 100%.

#### Cell Morphology Observation

The control group and the experimental groups each comprised 1.5 × 10^5^ cells and seeded them in a 6-well plate, added 10% fetal bovine serum in 2 mL and incubated overnight. Different concentrations of NC and BF were applied to experimental groups, accordingly. For simultaneous culture, 10% FBS RPMI-1640 media was added to the control group, and the cells were all incubated at 37 °C with 5% CO_2_ for 24 h to observe the morphological changes of the cells.

#### Western Blot Analysis

Cells were plated in 6-well plates, cultured for 24 h with various concentrations of NC and BF, centrifuged, and then washed three times with 1× PBS. One hundred microliters of cell lysate was then added to each well, and lysed on ice for 10 min. The supernatant was taken and used to quantify the expression levels of GAPDH (Beyotime, AF1186, 1:2000) and PARP (Beyotime, AF1657, 1:5000). Western blot analysis typically includes several steps, including protein separation based on molecular weight by sodium dodecyl sulfate-polyacrylamide gel electrophoresis (SDS-PAGE), electrophoretic transfer (Bole, PowerPac Basic) of proteins from the SDS-PAGE gels to polyvinylidene difluoride (PVDF) membranes (Millipore, ISEQ00010), and blocking the membrane with 5% skim milk. Transfer the membrane to the primary antibody solution, shake at 4 °C overnight, and then wash 3 times with PBST for 3–5 min. The membrane was then transferred to the secondary antibody (Abcom, ab6721, 1:10000) solution and shaked at room temperature for 1 h. Wash the membrane 3 times with PBST for 3–5 min.

#### Identification of Major Genes and Functional Enrichment Analysis

Drug chemical structure was downloaded from PubChem website, and uploaded to PharmMapper (http://lilab.ecust.edu.cn/pharmmapper/index.php) [14], which was used to predict target genes, and keep the number of matching genes for 300. The differentially expressed genes were downloaded from GEPIA (http://gepia.cancer-pku.cn/detail.php?clicktag = degenes) [15], and the screening parameters were Log2FC = 1, *q*-value = 0.01, and ANOVA method. Both elevated and reduced genes were selected [8]. GO (www.geneontology.org) analysis was performed to identify the biologic implications of the common genes. Fisher’s exact test was used to identify the significant GO terms with FDR-adjusted *P*-values. KEGG (http://www.kegg.jp) pathway analysis was performed to identify biologically important pathways associated with the common genes. Fisher’s exact test was used to select the significant pathways based on *P*-values (*P *< 0.05) and FDR (FDR < 0.27).

#### Statistics Analysis

SPSS 22.0 was used for data analysis and GraphPad Prism 6.0 was used for statistical mapping. For statistical analysis, Student’s t-test was used for parametric variables. One-way ANOVA followed by Dunnett’s test was used for comparisons among multiple groups. All tests were performed three times, and P < 0.05 was considered to indicate statistically significant differences.

## Results

### Detection of Inhibition Rate of Cell Proliferation

**Fig. 1:**
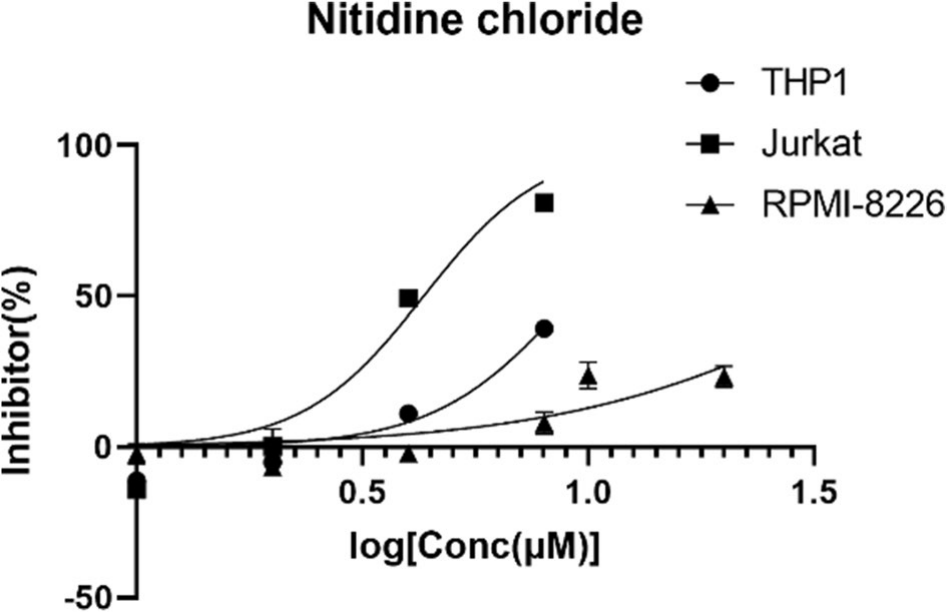
Proliferation inhibition rate of cells with different concentrations of NC

Following a 24-h treatment with NC at various concentrations in the cells, the inhibition rate increased as the drug concentration increased and was positively linked with the dosage (Fig. 1). Drug resistance to NC varied among various cell types. It was discovered that THP-1 cells (IC50 = 9.24 μm) and Jurkat cells (IC50 = 4.33 μm) showed different drug sensitivity to NC, whereas RPMI-8226 had a stronger drug resistance to NC (IC50 = 28.18 μm) (Table 1).

**Table 1:** Semi-inhibitory concentrations of NC and BF in different cells (IC50 of NC in different cells)

	THP-1	Jurkat	RPMI-8226
Nitidine chloride	9.24 μm	4.33 μm	28.18 μm
Bufalin	36.23 nM	26.71 nM	40.46 μm

**Fig. 2:**
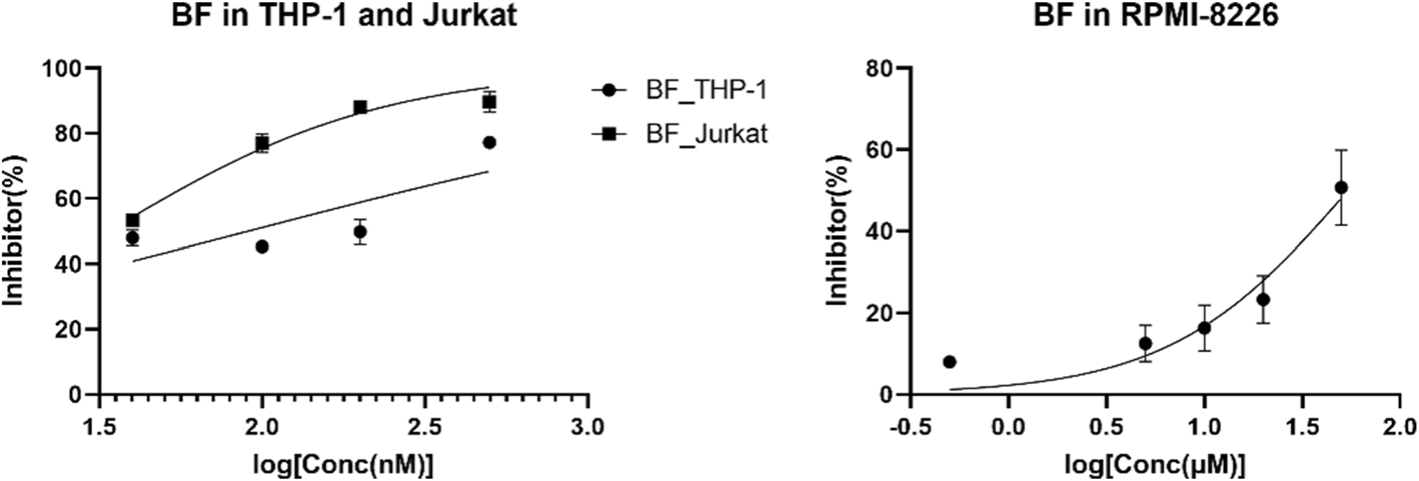
Proliferation inhibition rate of cells with different concentrations of BF (left, proliferation inhibition rate of THP-1 and Jurkat cells with different concentrations of BF; right, proliferation inhibition rate of RPMI-8226 cells at different concentrations of BF)

Interestingly, when different quantities of BF were applied in the same way to assess the inhibition rate of BF on cell proliferation, THP-1 cells and Jurkat cells were more sensitive to BF (Fig. 2A), with IC50 values of 36.23nM and 26.71nM, respectively (Table 1). However, with an IC50 of 40.46 μm, RPMI-8226 demonstrated greater drug resistance to BF (Fig. 2B) (Table 1).

### Morphological Observation of Cells

**Fig. 3:**
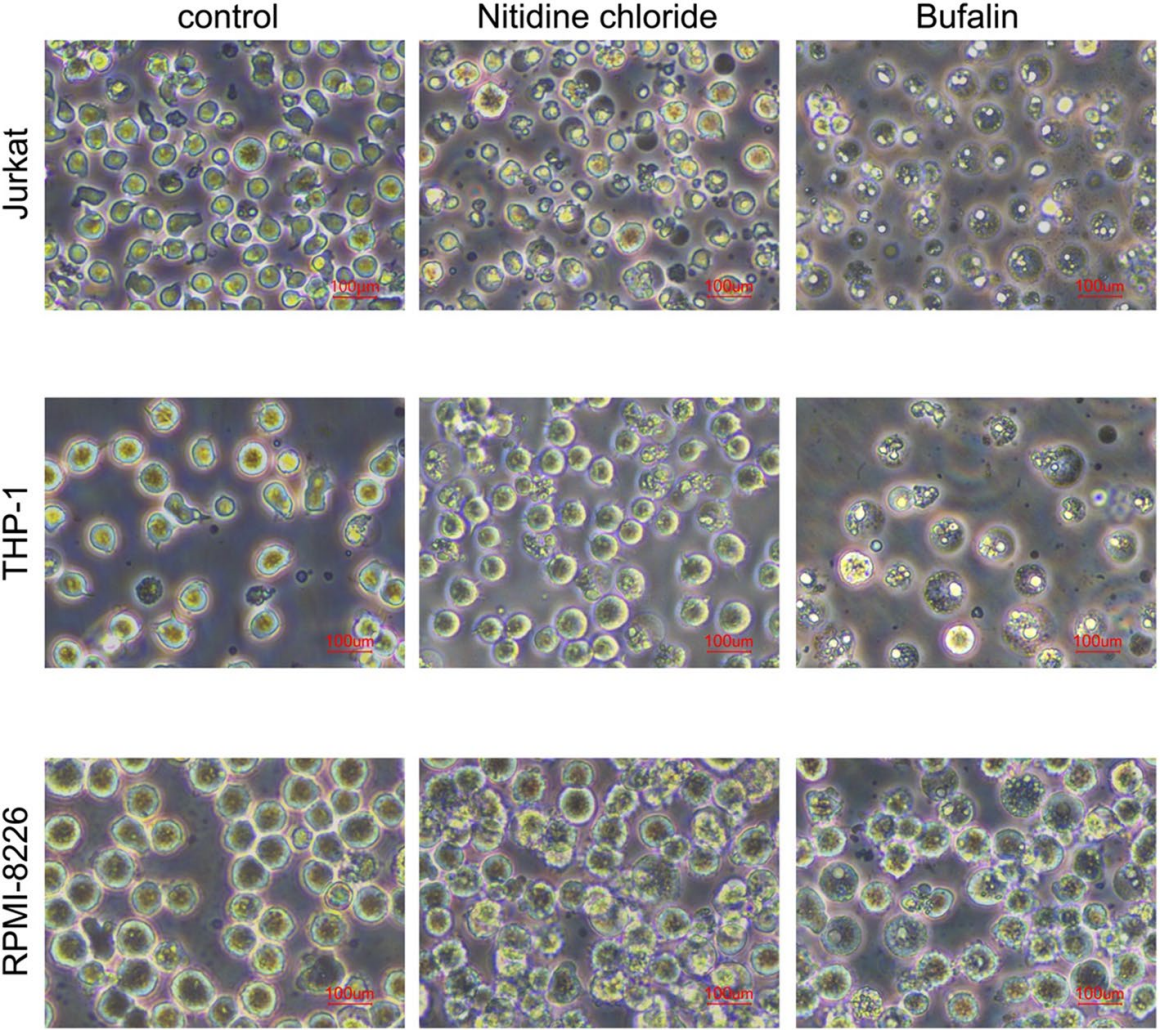
Picture of cell morphology (concentration of NC in Jurkat, THP-1, and RPMI-8226 is 4 μm, 4 μm, and 8 μm, respectively; concentration of BF in Jurkat, THP-1, and RPMI-8226 is 20nM, 20nM, and 4 μm, respectively)

We immediately employed a microscope to analyze the morphological changes of cells treated with various medications and varied dosages of medications in order to further validate whether NC and BF may suppress cell growth and trigger cell death. It was discovered that the morphological alterations in cells treated with NC and BF were different. RPMI-8226 cells’ cytoplasmic vacuoles grew and their cell borders were erratically shaped when the NC reached 8 μm (Fig. 3). THP-1 and Jurkat are manifested as nuclear contraction, some tumor cell membranes begin to rupture, contents are released, and cell proliferation slows down. After BF treatment, cells generally show cell swelling, cell membrane rupture, and content release (Fig. 3).

### Detection of Apoptosis-Related Proteins

**Fig. 4:**
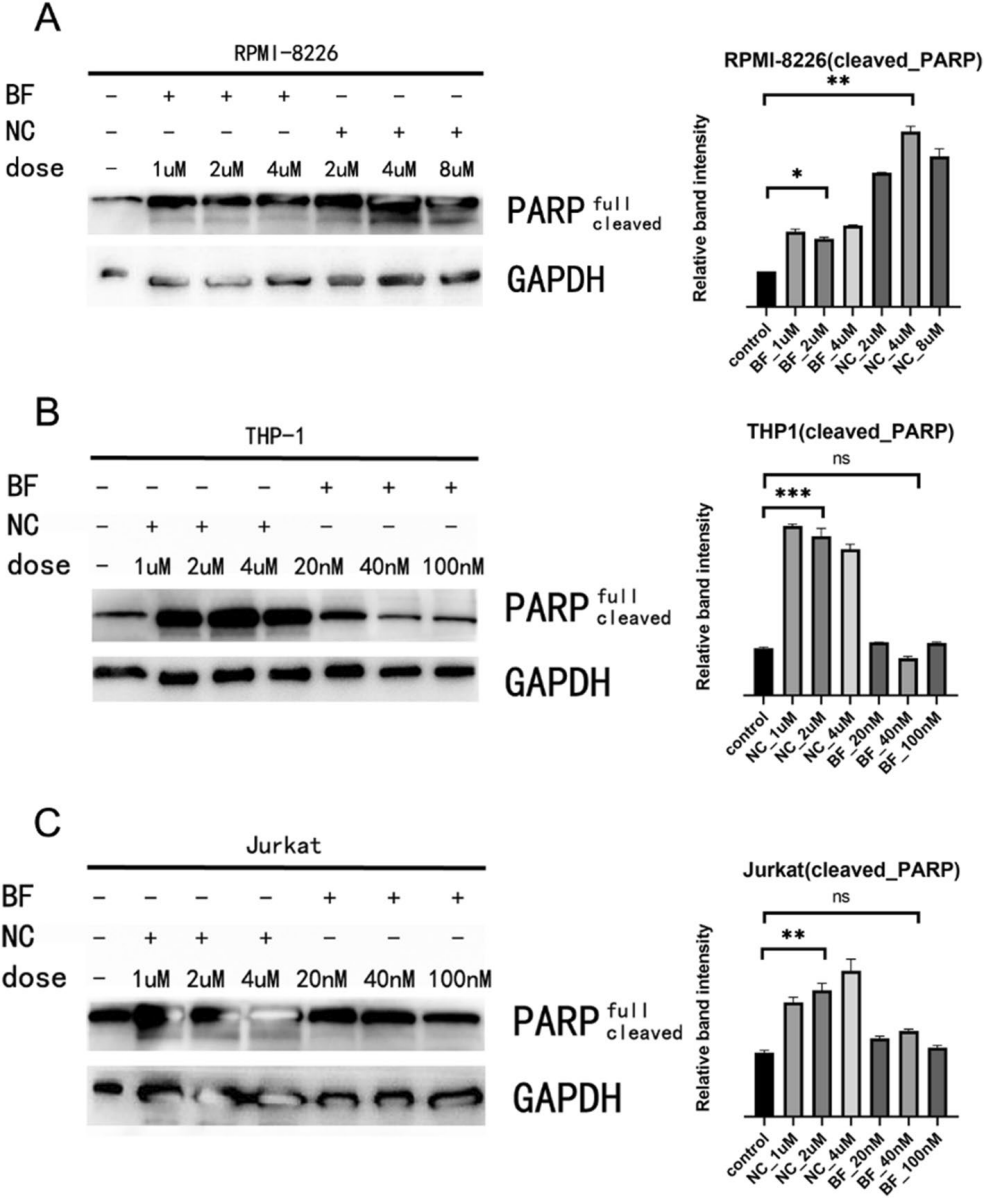
Western blot analysis of PARP protein (**A** RPMI-8226; **B** THP-1; **C** Jurkat)

In order to explore the molecular mechanism of PCD induced by NC and BF, we used western blot to detect the apoptosis-related protein PARP. At the same time, due to the large variation in IC50, different drug concentrations were chosen in order to avoid high concentrations of the drug causing severe toxic effects on the cells and affecting the test results. It was found that the cleavage of PARP could be obviously observed in cell PCD induced by NC, while the cleavage of PARP was not obvious in PCD induced by BF (Fig. 4). At the same time, different drug doses (1 μm, 2 μm, and 4 μm of BF in RPMI-8226; 2 μm, 4 μm, and 8 μm of NC in RPMI-8226; 1 μm, 2 μm, and 4 μm of NC in THP-1; 20nM, 40nM, and 80nM of BF in THP-1; doses of drug concentration in Jurkat is the same to THP-1) were chosen due to the significant range in IC50 in order to avoid excessive concentrations of the medication generating severe toxic effects on the cells and altering the result.

### Bioinformatics Analysis

**Fig. 5:**
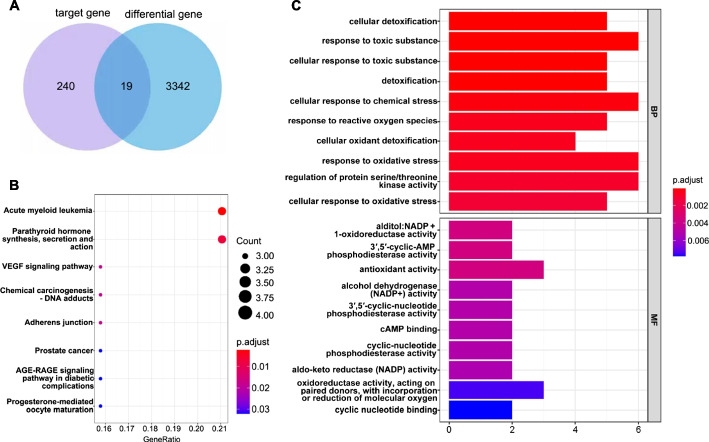
Result of functional enrichment analysis (**A** Venn diagram of BF target gene and LAML differential gene; **B** KEGG analysis of target common genes; **C** GO analysis of target common genes)

From the intersection of related genes in several databases, 19 important genes were discovered (Fig. 5A; Table 2). KEGG analysis using key genes showed that BF was involved in the regulation of pathways related to acute myeloid leukemia (Fig. 5B; Table 3). GO cluster analysis showed that BF was involved in cellular oxidative stress response, mediating cell apoptosis (Fig. 5C; Tables 4 and 5).

**Table 2:** Common genes of differential genes and target genes

Common genes	AKR1B1, AKR1C2, BACE1, CA2, CCNA2, DUSP6, EPHB4, FGFR1, GSTP1, ITGAL, MAPK1, NOS3, NQO1, PDE4B, PDE4D, PIM1, PLK1, PTGS2, WAS

**Table 3:** KEGG analysis

ID	Description	*P*-value
hsa05221	Acute myeloid leukemia	1.48E-05
hsa04928	Parathyroid hormone synthesis, secretion and action	9.03E-05
hsa04370	VEGF signaling pathway	0.000322062
hsa05204	Chemical carcinogenesis - DNA adducts	0.000511463
hsa04520	Adherens junction	0.000556296
hsa05215	Prostate cancer	0.001381078
hsa04933	AGE-RAGE signaling pathway in diabetic complications	0.001507968
hsa04914	Progesterone-mediated oocyte maturation	0.001596511

**Table 4:** GO analysis top10 (Biological processes, BP)

ID	Description	*P*-value
GO:1990748	Cellular detoxification	8.38E-08
GO:0009636	Response to toxic substance	9.19E-08
GO:0097237	Cellular response to toxic substance	1.13E-07
GO:0098754	Detoxification	2.09E-07
GO:0062197	Cellular response to chemical stress	8.23E-07
GO:0000302	Response to reactive oxygen species	2.29E-06
GO:0098869	Cellular oxidant detoxification	2.94E-06
GO:0006979	Response to oxidative stress	3.44E-06
GO:0071900	Regulation of protein serine/threonine kinase activity	6.21E-06
GO:0034599	Cellular response to oxidative stress	9.38E-06

**Table 5:** GO analysis (Molecular biological function, MF)

ID	Description	*P*-value
GO:0004032	Alditol:NADP + 1-oxidoreductase activity	6.67E-05
GO:0004115	3′,5′-cyclic-AMP phosphodiesterase activity	7.88E-05
GO:0016209	Antioxidant activity	9.14E-05
GO:0008106	Alcohol dehydrogenase (NADP+) activity	0.000211112
GO:0004114	3′,5′-cyclic-nucleotide phosphodiesterase activity	0.000254025
GO:0030552	cAMP binding	0.000254025
GO:0004112	Cyclic-nucleotide phosphodiesterase activity	0.000300844
GO:0004033	Aldo-keto reductase (NADP) activity	0.000351552
GO:0016705	Oxidoreductase activity, acting on paired donors, with incorporation or reduction of molecular oxygen	0.00057009
GO:0030551	Cyclic nucleotide binding	0.000662941

## Discussion

NC and BF, as traditional Chinese medicines, have been fully proven to have certain advantages in the treatment of tumors, chronic liver disease, kidney disease, diabetes, skin disease, and other diseases in clinical practice. However, due to the complexity of the important components, the pharmacological research and molecular mechanism of NC and BF remain unclear. Compared with BF, NC requires higher concentration to inhibit cell proliferation and promote PCD. Knowing how they work will enable more precise usage of them and lessen the toxicity of their effects.

In clinical practice, the benefits of NC and BF, two traditional Chinese medicines, have been thoroughly demonstrated in the treatment of cancers, chronic liver disease, kidney disease, diabetes, skin disease, and other illnesses. However, the pharmacological and molecular mechanism of NC and BF is still unknown because of the intricacy of the significant components. In our study, NC requires a higher concentration than BF in order to stop cell growth and encourage cell death. Understanding their mechanisms will allow for more accurate application and reduce the toxicity of their effects.

Pyroptosis, apoptosis, autophagy, and necroptosis are all forms of PCD, which is an active process of cell death that occurs when cells receive certain signals or are stimulated by certain factors in order to maintain the stability of the internal environment [16–18]. Pyroptosis is a recently discovered form of programmed cell death, which is characterized by the continuous expansion of cells until the cell membrane ruptures, leading to the release of cell contents and the activation of a strong inflammatory response [19]. It is now believed that apoptosis is a process of cell death that is triggered by pre-existing intracellular death programs, both in vivo and externally. Dozens of apoptosis-related genes are known, and they are divided into three categories according to their functions: apoptosis-inhibiting genes such as BCL-2 [20], apoptosis-promoting genes such as Bax and p53 [21, 22], and bidirectional regulatory genes such as c-myc and Fas/FasL pathways that deliver apoptotic signals and mediate apoptosis [23, 24]. Caspase-3 cleaves PARP and blocks the consumption of ATP during its activation, leading to a decrease in its ability to repair DNA and thus promoting the apoptotic process [25–27]. Apoptosis-inducing factors (toadstatin, amphotericin chloride, etc.) acting on leukemia cells may be transformed into apoptotic signals and, through different signaling pathways, eventually activate death genes, leading to apoptosis. The process of apoptosis signaling is regulated by a variety of genes in a precise and tightly regulated manner.

It is interesting to note that BF-treated cells showed a general cell swelling phenomenon. This phenomenon may be related to the fact that BF interferes with the sodium-potassium pump’s normal operation, which disrupts both internal and exterior cellular homeostasis and causes cell swelling [28]. It is necessary to confirm in later trials whether pyroptosis or the modulation of oxidative stress from the inside out is the real cause of subsequent cell death. Another issue of interest is that NC and BF undoubtedly have some inhibitory effect on tumor cell proliferation, and the inhibitory effect is more pronounced with increasing doses. However, it is not clear why there is a difference in the mode of cell death induced by these two and why they have different killing effects on different types of hematological tumor cells.

Regulators and effectors in different cell death pathways are highly likely to be therapeutic targets for tumor cells, and they may form the basis of translational medicine that promises to improve clinical care for patients with hematologic tumors [12, 29, 30]. Given the complex etiology of circulatory diseases, in which multiple cell death mechanisms are often combined with other cellular processes and thus jointly drive pathological progression, it seems that the induction of more than one cell death program is more promising as a subsequent therapeutic direction for this type of disease.

In summary, our study shows that NC and BF can inhibit tumor cell proliferation and mediate PCD and that different cells have different sensitivity to the drugs, but further exploration of the specific molecular mechanisms is needed to develop the drugs for clinical application and to make targeted dose adjustments or drug combinations to improve drug efficacy and reduce drug side effects.

## Data Availability

The data and materials of this experiment are available.
